# The Heredity Factor in Disease

**Published:** 1901-09-21

**Authors:** 


### The Heredity Factor in Disease.

The almost universal knowledge of the general principles underlying the theories concerning the origin of species has undoubtedly caused thinkers to devote their attention largely to the part played by hereditary disposition both in health and disease and both in normal and abnormal physical and mental conditions. As we advance from the lower to the higher manifestations of nature we must notice that the regular order and method becomes more irregular, diverse, and unexpected. One crystal of a salt is exactly similar to another, and behaves with uniformity under similar conditions, but no two plants or animals are exactly identical. When we reach the highest manifestations with which we are acquainted, the workings of the mind of man, the diversity is still greater, and we cannot assure that two brothers, reared under exactly similar circumstances, will not show absolutely opposite dispositions and characteristics.

On the other hand, the physical likeness in one family is frequently well marked, and we are all familiar with numerous instances where some particular disease ravaged a whole clan. But in the scientific and systematic classification of heredity as a cause of disease, the mathematical difficulties are insurmountable. We have several factors to consider. First, the active cause which is usually a germ, or some abnormal chemical condition of the blood. Secondly, the environment of the individual. Under the heading of environment we must place the effects of localities, occupations and sanitary conditions of the home. Thirdly, the powers of resistance may have been weakened by disease or habits prejudicial to health. Lastly, we have the hereditary factor.

In examining a patient we ask carefully for the family history, and, provided he knows enough of the diseases of his relations, in all probability we shall discover an hereditary tendency to phthisis, cancer, gout, alcoholism, insanity, or any other disorder of which we may inquire. Before this evidence is of any value it has to be carefully balanced against the laws of probabilities, when we shall discover that, instead of it being an alarming fact that some cousin died of consumption, it would be a very extraordinary thing if no relation had succumbed to that disease.

Again, supposing a person with an undoubted hereditary tendency to phthisis—where, for example, a parent and one or two brothers or sisters have died from that disease—still it may be possible, by carefully choosing his environment, to strengthen the resistance of his organs to the attacks of the tubercle bacillus and he may escape the disease altogether. Or again by careful isolation it is conceivable that he would not come into contact with enough germs to occasion infection. These three factors, the active cause, the powers of resistance of the tissues and the environment play so great a part in the determination of tubercular diseases that in all probability the heredity factor will speedily be recognised as but of secondary and subordinate consequence.

In cancer the hereditary tendency has never been regarded as of such great importance, but we know that if the powers of resistance of the tissues are exhausted by continual irritation, the invasion of the epithelial cells may follow. If the growth of cancer is due to the stimulation of some organism then it is easy to understand that if the powers of resistance of the healthy cells are reduced, they are no longer able to withstand the attack.

When we consider gout the difficulty of attaching any importance to hereditary tendency becomes much greater still. In the first place nearly everyone has or has had pains in the joints which were loosely called gout, rheumatic gout, or rheumatism before the distinction between rheumatoid arthritis was recognised. Again, when we remember its pathology, it becomes extremely difficult to reconcile the presence of an abnormal substance in the blood with an hereditary tendency, particularly when we know that it is due to imperfect digestion and assimilation of the food stuffs.

Mental diseases, again, may be hereditary; but here, also, the law of probabilities must be considered. There is a disposition to link up insanity, crime, and even genius—whatever the term may mean—and regard them all as due to some abnormal condition of the mind. Further, some writers have taken the histories of several families of criminals and proved their descent from persons of unsound mind. The results are certainly very startling, but let us take the history of *every* drunkard, thief, and prostitute and then see the proportion who have normal relations to those who have not.

